# Behavioral and Neurochemical Deficits in Aging Rats with Increased Neonatal Iron Intake: Silibinin’s Neuroprotection by Maintaining Redox Balance

**DOI:** 10.3389/fnagi.2015.00206

**Published:** 2015-10-28

**Authors:** Hanqing Chen, Xijin Wang, Meihua Wang, Liu Yang, Zhiqiang Yan, Yuhong Zhang, Zhenguo Liu

**Affiliations:** ^1^Department of Neurology, Xinhua Hospital Affiliated to Shanghai Jiao Tong University School of Medicine, Shanghai, China; ^2^School of Biotechnology and Food Engineering, Hefei University of Technology, Hefei, China; ^3^Shanghai Laboratory Animal Center, Chinese Academy of Sciences, Shanghai, China; ^4^Department of Neurology, Shanghai Tenth People’s Hospital, Tongji University, Shanghai, China

**Keywords:** aging, Parkinson’s disease, dopamine, behavior, MDA, GSH, silibinin

## Abstract

Aging is a critical risk factor for Parkinson’s disease. Silibinin, a major flavonoid in *Silybum marianum*, has been suggested to display neuroprotective properties against various neurodegenerative diseases. In the present study, we observed that neonatal iron (120 μg/g body weight) supplementation resulted in significant abnormality of behavior and depletion of striatal dopamine (DA) in the aging male and female rats while it did not do so in the young male and female rats. No significant change in striatal serotonin content was observed in the aging male and female rats with neonatal supplementation of the same dose of iron. Furthermore, we found that the neonatal iron supplementation resulted in significant increase in malondialdehyde (MDA) and decrease in glutathione (GSH) in the substantia nigra (SN) of the aging male and female rats. No significant change in content of MDA and GSH was observed in the cerebellum of the aging male and female rats with the neonatal iron supplementation. Interestingly, silibinin (25 and 50 mg/kg body weight) treatment significantly and dose-dependently attenuated depletion of striatal DA and improved abnormality of behavior in the aging male and female rats with the neonatal iron supplementation. Moreover, silibinin significantly reduced MDA content and increased GSH content in the SN of the aging male and female rats. Taken together, our results indicate that elevated neonatal iron supplementation may result in neurochemical and behavioral deficits in the male and female rats with aging and silibinin may exert dopaminergic neuroprotection by maintaining redox balance.

## Introduction

Parkinson’s disease (PD) is a common neurodegenerative disorder characterized by cardinal features, including resting tremor, rigidity, slowness of movement, and postural instability. It is due to decreased dopamine (DA) content in the striatum as a result of progressive and selective degeneration of dopaminergic neurons in the substantia nigra (SN). In recent years, more and more studies have demonstrated that the causes of PD are multifactorial, including aging, exposure to environmental toxins, immune/inflammatory factors, genetic predisposition, and innate characteristics of the nigrostriatal dopaminergic system in the brain (Olanow and Tatton, 1999; Kidd, 2000; Gao et al., 2003; Wang et al., 2005a,b, 2007a,b, 2011; Zhang et al., 2011; Connolly and Lang, 2014). For a long time, because it is difficult to obtain aging animals, young animals are often applied to PD research. However, PD is a neurodegenerative disease that is closely associated with aging. Among those factors that have the potential to play a role in idiopathic PD, aging is a critical risk factor for this disease (Yankner et al., 2008; Gureviciene et al., 2009; Hindle, 2010). Epidemiological survey also exhibits that PD affects about 1–2% of the population over the age of 65, and incidence and prevalence further increase with advancing age (Von Campenhausen et al., 2005; Yankner et al., 2008; Hindle, 2010). So, compared with young animals, it is more significant that aging animals are employed into PD research. With further insight into aging and PD, increasing importance is being attached to develop effective therapy strategies for PD through intervening aging-related changes and deficits using aging animals (Dauer and Przedborski, 2003; Connolly and Lang, 2014).

Among the exposure of human to environmental chemicals, the metals/metalloids may be the leading toxic agents detected in the environment (Migliore and Coppedè, 2009; Chege and McColl, 2014). Iron is one of the essential trace metals for human body and has been reported to be involved in electron transfer, oxygen transport, neurotransmitter synthesis, and myelin production in the central nervous system (CNS) (Stankiewicz et al., 2007). Insufficient iron intake can result in iron-deficient anemia (Anand et al., 2014). In addition, in early human life, severe iron deficiency can lead to impaired brain development (Lozoff and Georgieff, 2006; Radlowski and Johnson, 2013). Based on these considerations, it has been recommended that while human milk is the preferred food source for all infants, children who are not breast-fed or partially breast-fed should be provided with an iron-fortified formula. Therefore, it is of interest to explore the effect of neonatal dietary iron feeding on the CNS in aging process. Although a previous study conducted by Kaur et al. (2007) has suggested that increased neonatal iron intake in mice can induce an aging-related dopaminergic neurodegeneration, which is similar to PD, it remains unclear about the effect of increased neonatal iron treatment on motor behavior and neurotransmitters of aging male and female animals. Silibinin, a flavonoid isolated from the herb milk thistle (*Silybum marianum* L.), has been used clinically for thousands of years in China and Europe as an anti-hepatotoxic agent to treat liver diseases, especially alcoholic liver disease (Guigas et al., 2007; Brandon-Warner et al., 2010). Silibinin has also been suggested to display neuroprotective properties against various neurodegenerative diseases (Lu et al., 2009; Wang et al., 2012). However, it is not clear whether silibinin is neuroprotective against aging-related neurodegeneration in male and female animals.

In the present study, we examined the effect of increased neonatal dietary iron feeding on behavior and neurotransmitters in male and female rats with aging. Importantly, we investigated the effect of silibinin on behavior and neurotransmitters in aging male and female rats with increased neonatal iron intake and the underlying mechanism.

## Materials and Methods

### Animals and Treatment

All animals were purchased from Sino-British SIPPR/BK Lab Animal Ltd., Shanghai, China. During the course of the experiments, all animals were treated in strict accordance with the guidelines of the US National Institutes of Health for the Care and Use of Laboratory Animals (NIH Publication No. 85-23, revised 1996) and the Protocols for Animal Experimentation of Shanghai Jiao Tong University School of Medicine. All efforts were made to minimize the number of animals used and their suffering. Sprague-Dawley rat pups were fed either saline vehicle or carbonyl iron daily by oral gavage from days 10 to 17 post-partum. Based on the previous studies (Schröder et al., 2001; Kaur et al., 2007), the rat pups were fed with increased iron (120 μg/g body weight). Rats were divided into young and aging groups. The rats in young and aging groups were aged to 200 and 600 days, respectively, and behavior tests were performed on the rats. Then, rats were sacrificed for further experiments. From the age of 540 days, some aging rats was administered with silibinin (25 and 50 mg/kg body weight) every second day orally through an oral gavage needle before scarification.

### Behavior Tests

Rotarod and open field tests were carried out to evaluate animal behavior during the light period. The basic requirements for rotarod test were composed of a power source, a roller, and four separators that divided the roller into equal-sized compartments. After receiving training, rats were tested for three times at each rotarod speed of 5, 10, and 15 rotations per minute (rpm). The latency time to fall was recorded for each test. For locomotor activity, each rat was placed into an open field chamber, which is made of wood covered with impermeable formica. The chamber had a white floor (100 cm × 100 cm) with it divided into 25 squares (20 cm × 20 cm) and 50 cm high walls. Before testing, each animal was placed in the open field center and acclimatized for 10 min. Rat motor behavior was recorded for 30 min. The following parameters were counted: (1) crossing number, defined as entering of another square with all four paws and (2) rearing number, defined as rearing with and without wall contact (standing only on hind legs).

### Neurochemical Analysis

High-performance liquid chromatography (HPLC), equipped with electrochemical detector (ECD), was used to determine neurotransmitter content in the rat striata (McNaught et al., 2004). Briefly, rat striata were dissected on ice and weighed. Then, the striata were homogenized (10% weight/volume) by sonication in ice-cold homogenization buffer containing perchloric acid (0.1 mol/L). 3,4-Dihydroxybenzylamine is applied as the internal standard. Obtained samples were centrifuged at 25,000 g and 4°C for 10 min. The collected supernatants were used for the determination of DA and serotonin (5-HT) content by HPLC-ECD, which is equipped with a column of 5 μm spherical C18 particles. The mobile phase was composed of 0.1 M phosphate buffer (pH 2.6) containing 2.5% methanol, 0.2 mM octanesulfonic acid, and 4.5% acetonitrile. The content of each neurotransmitter was expressed as nanograms per gram equivalent of striatal tissue.

### Determination of Malondialdehyde (MDA) and Glutathione (GSH)

The levels of MDA and GSH were measured according to the manufacturer’s instruction by commercially available kits (Cayman Chemical Co., Ann Arbor, MI, USA). For MDA assay, thiobarbituric acid (TBA) reacts with MDA in acidic medium at elevated temperature to form TBA reactive product. SN tissue was homogenized (10% weight/volume) with radioimmunoprecipitation assay (RIPA) homogenizing buffer containing a protease inhibitor. Obtained samples were centrifuged at 1,600 g and 4°C for 10 min. Then, supernatants were used for analysis. Samples or standards (100 μl) were added to 100 μl of sodium dodecyl sulfate solutions followed by the addition of 4 ml of color reagents (530 mg of TBA with 50 ml of diluted TBA acetic acid solution and 50 ml of diluted TBA sodium hydroxide). Obtained solutions were boiled for 1 h followed by the incubation in ice bath for 10 min to stop reaction. Then, solutions were centrifuged at 1,600 g and 4°C for 10 min. The absorbance values of the samples were read at 540 nm using a microplate reader. GSH determination was based on the enzymatic recycling method. In brief, 100 μl of supernatant from SN homogenate was deproteinated by the addition of 100 μl metaphosphoric acid reagent (5 g of metaphosphoric acid in 50 ml water). Then, triethanolamine reagent (50 μl/ml, 4 M) was added to the solution and immediately vortexed. Samples or standards (50 μl) were pipetted into the designated wells. This was followed by the addition of 150 μl of freshly prepared Assay Cocktail consisting of 11.25 ml of MES Buffer (0.4 M 2-(N-morpholino) ethanesulphonic acid, 2 mM EDTA, and 0.1 M phosphate), 0.45 ml of reconstituted Cofactor Mixture (a lyophilized powder of NADP^+^ and glucose-6-phosphate reconstituted with 0.5 ml of water), 2.1 ml of reconstituted Enzyme Mixture (glutathione reductase and glucose-6-phosphate dehydrogenase reconstituted in 2 ml MES buffer), 2.3 ml of water, and 0.45 ml of reconstituted DTNB (5,5′-dithio-*bis*-(2-nitrobenzoic acid)). Following incubation of 25 min, the absorbance value was read at 405 nm using a microplate reader.

### Statistical Analysis

Data were expressed as the mean ± SEM. Differences were determined by two-tailed Student’s *t*-test for comparison between two groups and an analysis of variance (ANOVA) and Bonferroni *post hoc* test for comparison between more than two groups. Normality of sample distribution and homogeneity of variances were tested before each ANOVA. A value of *p* <0.05 was considered statistically significant.

## Results

### Increased Neonatal Iron Intake Resulted in Significant Behavior Abnormality and Striatal DA Depletion in Male and Female Rats with Aging

**Figure 1:**
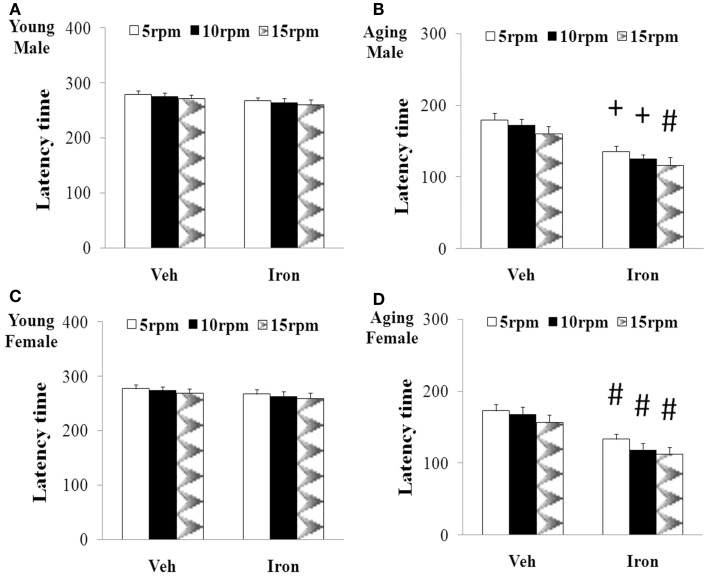
**Increased neonatal iron intake resulted in significant behavior abnormality (rotarod test) in male (A,B) and female (C,D) rats with aging [(A,C): young; (B,D): aging]**. Results are expressed as mean ± SEM. *N* = 8–10. ^#^*p* < 0.01, ^+^*p* < 0.005, compared with the aging rats treated with Veh. Veh, vehicle.

**Figure 2:**
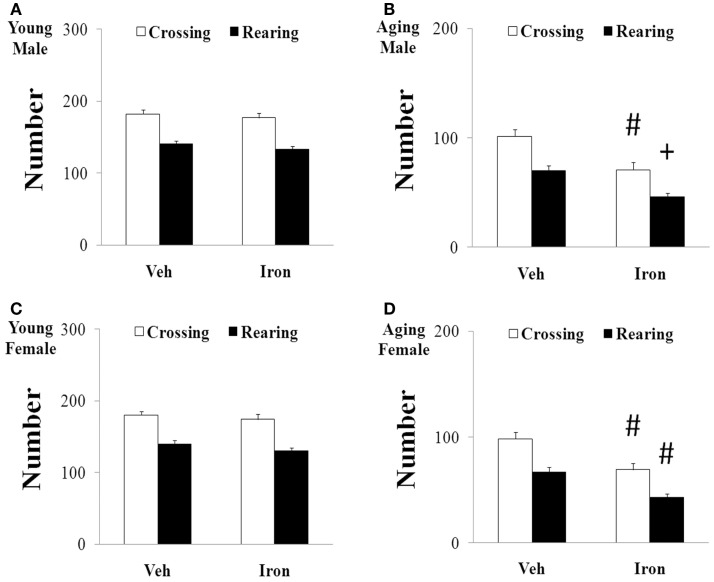
**Increased neonatal iron intake resulted in significant behavior abnormality (open-field test) in male (A,B) and female (C,D) rats with aging [(A,C): young; (B,D): aging]**. Results are expressed as mean ± SEM. *N* = 8–10. ^#^*p* < 0.01, ^+^*p* < 0.005, compared with the aging rats treated with Veh. Veh, vehicle.

**Figure 3:**
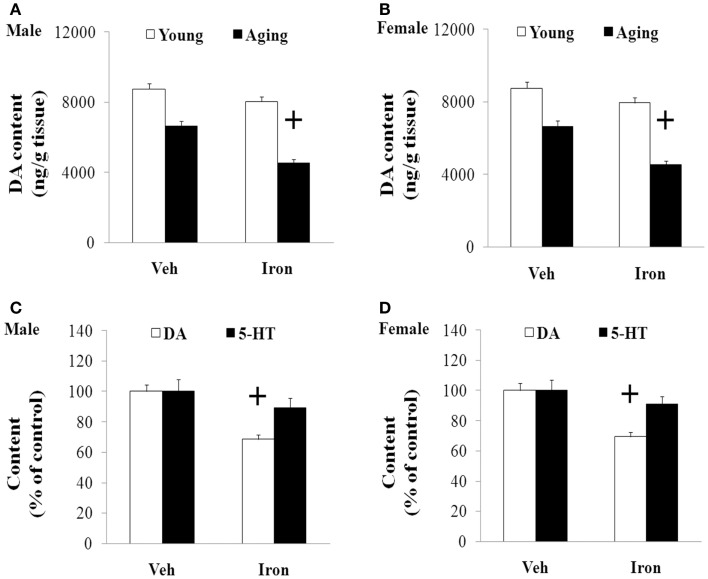
**Increased neonatal iron intake resulted in significant striatal DA depletion in male (A,C) and female (B,D) rats with aging**. Results are expressed as mean ± SEM. *N* = 8–10. ^+^*p* < 0.005, compared with the aging rats treated with Veh. DA, dopamine; 5-HT, serotonin; Veh, vehicle.

Rotarod and open field tests were carried out to evaluate the effect of neonatal iron intake on motor behavior of young and aging rats (male and female). As shown in Figures 1 and 2, neonatal iron (120 μg/g body weight) intake did not lead to significant change in behavior of the young male and female rats in comparison with the vehicle-treated rats. However, significant decreases in latency time [aging male rats: *p* < 0.005 (5 and 10 rpm), *p* < 0.01 (15 rpm); aging female rats: *p* < 0.01 (5, 10, and 15 rpm)] and the number of crossing and rearing [aging male rats: *p* < 0.01 (crossing number), *p* < 0.005 (rearing number); aging female rats: *p* < 0.01 (crossing number and rearing number)] were observed in the aging male and female rats with neonatal intake of the same dose of iron in comparison with the vehicle-treated rats (Figures 1 and 2). In agreement with behavior tests, the neonatal iron intake did not lead to significant depletion of striatal DA in the young male and female rats in comparison with the vehicle-treated rats (Figures 3A,B). However, significant decrease in striatal DA content (*p* < 0.005) was observed in the aging male and female rats with neonatal iron intake compared with the vehicle-treated rats (Figures 3A,B). Although the neonatal iron intake significantly reduced the content of striatal DA in the aging male and female rats compared with the vehicle-treated rats, no significant change in the content of striatal 5-HT was observed in the aging male and female rats with the neonatal iron intake in comparison with the vehicle-treated rats (Figures 3C,D).

### Increased Neonatal Iron Intake Resulted in Significant MDA Increase and GSH Decrease in Male and Female Rats with Aging

**Figure 4:**
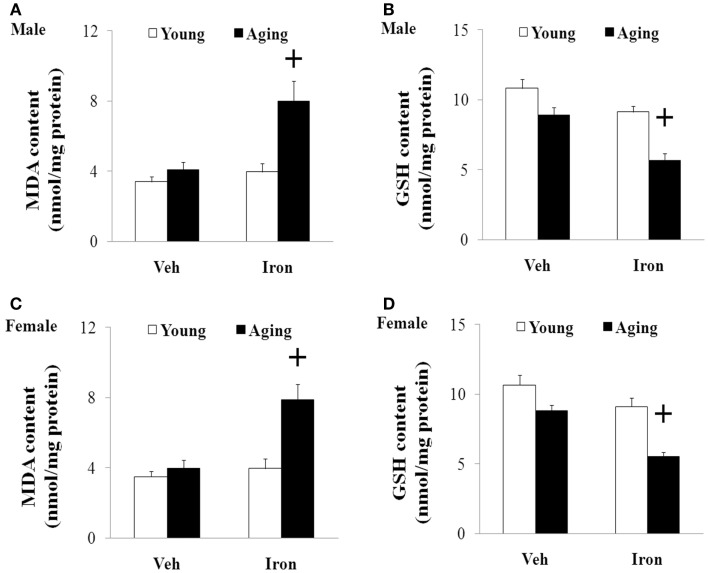
**Increased neonatal iron intake resulted in significant MDA (A,C) increase and GSH (B,D) decrease in the SN of male (A,B) and female (C,D) rats with aging**. Results are expressed as mean ± SEM. *N* = 8–10. ^+^*p* < 0.005, compared with the aging rats treated with Veh. MDA, malondialdehyde; GSH, glutathione; SN, substantia nigra; Veh, vehicle.

**Figure 5:**
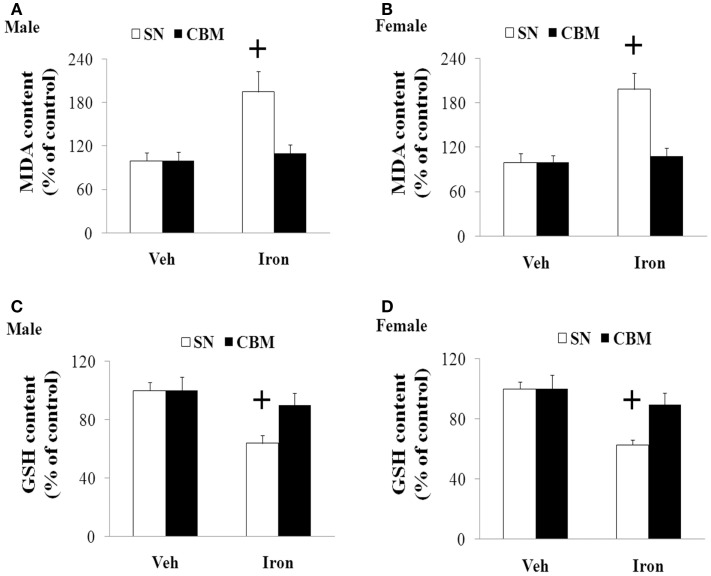
**Effect of increased neonatal iron intake on MDA (A,B) increase and GSH (C,D) content in the CBM of aging male (A,C) and female (B,D) rats with aging**. Results are expressed as mean ± SEM. *N* = 8–10. ^+^*p* < 0.005, compared with the aging rats treated with Veh. MDA, malondialdehyde; GSH, glutathione; SN, substantia nigra; CBM, cerebellum; Veh, vehicle.

We further investigated the effect of neonatal iron intake on the content of MDA and GSH in the SN of the young and aging rats (male and female). As shown in Figure 4, the neonatal iron intake did not result in significant change in the content of MDA and GSH in the SN of the young male and female rats compared with the vehicle-treated rats. However, significant increase in MDA content (*p* < 0.005) and decrease in GSH content (*p* < 0.005) were observed in the SN of the aging male and female rats with neonatal intake of the same dose of iron in comparison with the vehicle-treated rats. Although the neonatal iron intake significantly increased MDA content and decreased GSH content in the SN of the aging male and female rats compared to the rats treated with vehicle, no significant change in the content of MDA and GSH was observed in the cerebellum (CBM) of the aging male and female rats with the neonatal iron intake in comparison with the vehicle-treated rats (Figure 5).

### Silibinin Significantly and Dose-Dependently Diminished Striatal DA Depletion and Improved Behavioral Abnormality in Aging Male and Female Rats with Increased Neonatal Iron Intake

**Figure 6:**
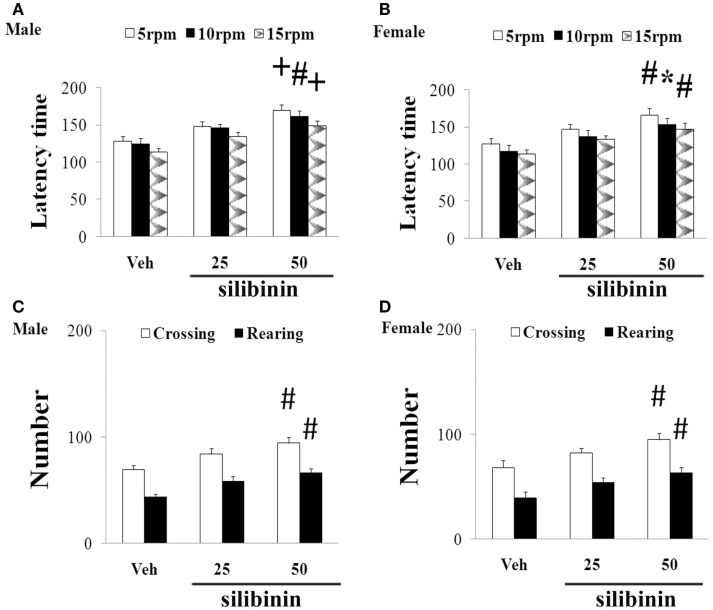
**Effect of silibinin treatment on motor behavior of aging male (A,C) and female (B,D) rats with increased neonatal iron intake in rotarod test (A,B) and open-field test (C,D)**. Results are expressed as mean ± SEM. *N* = 8–10. *p < 0.05, ^#^*p* < 0.01, and ^+^*p* < 0.005, compared with the aging rats treated with Veh. Veh, vehicle.

**Figure 7:**
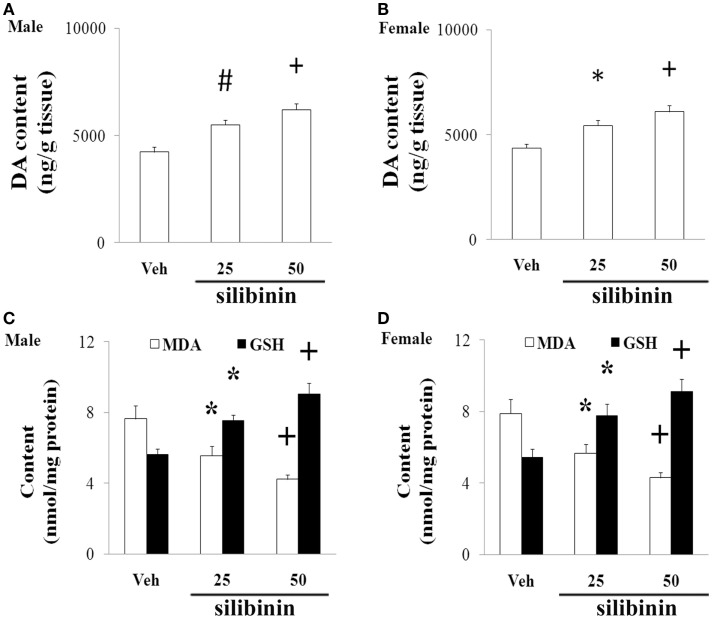
**Effect of silibinin treatment on striatal DA content and SN MDA and GSH content in aging male (A,C) and female (B,D) rats with increased neonatal iron intake**. Results are expressed as mean ± SEM. *N* = 8–10. *p < 0.05, ^#^*p* < 0.01, and ^+^*p* < 0.005, compared with the aging rats treated with Veh. DA, dopamine; Veh, vehicle; MDA, malondialdehyde; GSH, glutathione.

We further investigated the effect of silibinin (25 and 50 mg/kg body weight) treatment on striatal DA depletion and behavioral abnormality in the aging rats (male and female) with increased neonatal iron intake. As shown in Figure 6, although silibinin treatment at the dose of 25 mg/kg body weight did not significantly change motor behavior in the aging male and female rats with the neonatal iron intake compared with the vehicle-treated rats, motor behavior of the aging male and female rats with increased neonatal iron intake was significantly improved [aging male rats: *p* < 0.005 (5 and 15 rpm), *p* < 0.01 (10 rpm), *p* < 0.01 (crossing and rearing number); aging female rats: *p* < 0.01 (5 and 15 rpm), *p* < 0.05 (10 rpm), *p* < 0.01 (crossing and rearing number)] after silibinin administration at the dose of 50 mg/kg body weight. In agreement with behavior tests, neurochemical analysis also demonstrated that silibinin administration at the doses of 25 and 50 mg/kg body weight significantly diminished depletion of striatal DA in the aging male and female rats with increased neonatal iron intake compared with the vehicle-treated rats [aging male rats: *p* < 0.01 (25 mg/kg), *p* < 0.005 (50 mg/kg); aging female rats: *p* < 0.05 (25 mg/kg), *p* < 0.005 (50 mg/kg)] (Figures 7A,B).

### Silibinin Significantly Reduced MDA Content and Increased GSH Content in the SN of the Aging Male and Female Rats with Increased Neonatal Iron Intake

We finally examined the effect of silibinin treatment on MDA and GSH levels in aging rats with increased neonatal iron intake. As shown in Figures 7C,D, at the doses of 25 and 50 mg/kg body weight, silibinin administration significantly reduced the content of MDA and increased the content of GSH in the SN of the aging male and female rats with the increased neonatal iron intake compared with the vehicle-treated rats [*p* < 0.05 (25 mg/kg) and *p* < 0.005 (50 mg/kg)].

## Discussion

Many evidences have demonstrated that aging is a critical risk factor for idiopathic PD in recent years (Yankner et al., 2008; Gureviciene et al., 2009; Hindle, 2010). PD is rarely occurred before the age of 50 years for humans. Its incidence and prevalence increase with aging. Also, aging people gradually exhibit some characteristics of PD, including Lewy bodies, striatal DA decrease, and motor signs, which are similar to those seen in PD. In the mice with increased neonatal dietary iron feeding, Kaur et al. (2007) found increased SN iron level at the age of 3 months, increased oxidative stress markers and reduced striatal DA level at 12, 16, and 24 months, but not at 2 months, when compared with the control mice. They also found significant reduction in the number of tyrosine hydroxylase-immunoreactive neurons in the iron-fed animals in comparison with the control animals at the age of 24 months, but not at 2, 12, and 16 months. In the present study, our results indicated that increased neonatal iron (120 μg/g body weight) feeding induced significant abnormality of behavior and depletion of striatal DA in the aging male and female rats while it did not do so in the young male and female rats. Moreover, no significant change in the content of striatal 5-HT was observed in the aging male and female rats with neonatal intake of the same dose of iron. These results mentioned above suggested that elevated neonatal iron intake alone, given enough time, might lead to some features of PD. In addition, there was no significant change in striatal 5-HT content in the aging male and female rats, indicating the selectivity of neurotoxicity. Our results show that elevated neonatal iron supplementation has long-lasting effects, and it possibly represent a novel risk factor for aging-related dopaminergic neurodegeneration. The possible neurotoxicity of increased neonatal iron supplementation observed in the present work should be taken into consideration and certainly warrants further studies in humans especially because it affects the CNS in aging process. It is of interest for the development of effective treatments to diminish aging-related dopaminergic neurotoxicity as a consequence of elevated neonatal iron supplementation in aging male and female animals.

Silibinin is the major active component of silymarin extracted from milk thistle. By employing a ThT assay and electron microscopic imaging, Yin et al. (2011) identified that silibinin appears to act as a novel inhibitor of amyloid β (Aβ) aggregation and this effect showed dose-dependency. They also observed that silibinin protected SH-SY5Y cells from injuries caused by Aβ1-42-induced oxidative stress by decreasing H_2_O_2_ production in Aβ1-42-stressed neurons. Tota et al. (2011) showed that treatment with silibinin prevented streptozotocin-induced memory loss in mice. They further demonstrate that beneficial effect of silibinin in animals is contributed to improvement in brain energy metabolism and cholinergic function. Silibinin was also demonstrated to attenuate cognitive deficits and decreases of DA and 5-HT induced by repeated methamphetamine treatment (Lu et al., 2010). Jung et al. (2014) observed that silibinin administration attenuated MPP^+^-induced neurotoxicity in the SN in a dose-dependent manner. They also observed that increased levels of inflammatory molecules, such as tumor necrosis factor-alpha, interleukin-1 beta, and inducible nitric oxide synthase by MPP^+^ treatment, were attenuated by treatment with silibinin. Moreover, Geed et al. (2014) showed that silibinin pretreatment diminished biochemical and behavioral changes induced by intrastriatal MPP^+^ injection in rats. In the present study, we found that silibinin treatment significantly improved motor abnormality and diminished striatal DA depletion of the aging male and female rats with increased neonatal iron intake, indicating the potential dopaminergic neuroprotection of silibinin in PD.

Oxidative stress is demonstrated to be involved in aging process and cell injury (Bodhinathan et al., 2010; Argüelles et al., 2011; Cui et al., 2012; Sykora et al., 2013; Kamarudin et al., 2014). Redox imbalance may play an important role in various neurodegenerative diseases (Campos et al., 2014; Lee et al., 2014; Navarro-Yepes et al., 2014; Rivas-Arancibia et al., 2015; Wang et al., 2015). Content of MDA, lipids, and cholesterol hydroperoxides was observed to be increased in PD (Dexter et al., 1989, 1994). MDA, as the most cytotoxic aldehyde derived from the process of lipid peroxidation, reflects oxidative damage to lipids, and it has been observed to be significantly elevated in the SN of PD patients in comparison with other brain regions and control tissues (Esterbauer et al., 1991; Ross et al., 1998; Navarro-Yepes et al., 2014). GSH is an antioxidant existed in animal cells. It is an essential ubiquitous thiol tripeptide. Through reacting with free radicals and decreasing superoxide radicals, hydroxyl radicals, and peroxynitrites, GSH was shown to play an important role in cell protection. GSH has also been demonstrated to be involved in various biological processes, including DNA synthesis and repair, protein synthesis, enzymatic reaction, and cellular immunity (Cui et al., 2012; Navarro-Yepes et al., 2014). GSH decrease is exhibited in the SN of some PD animal models and PD patients (Ferraro et al., 1986; Navarro-Yepes et al., 2014). In the present study, we observed that increased neonatal iron intake resulted in significant MDA increase and GSH reduction in the SN of the male and female rats with aging. However, no significant change in the content of cerebellar MDA and GSH was observed in the aging male and female rats with increased neonatal iron intake. We also observed that silibinin significantly decreased the content of MDA and increased the content of GSH in the aging male and female rats with increased neonatal iron intake. Our results indicate that redox imbalance may be involved in dopaminergic neurodegeneration of aging male and female rats with increased neonatal iron intake and recovering balance of redox in the tissue microenvironment may be one of the most likely mechanisms by which silibinin exerts neuroprotective effects in aging male and female rats with increased neonatal iron intake.

In conclusion, our study suggests that elevated neonatal iron supplementation may induce PD-like neurochemical and behavioral deficits with aging and silibinin may be neuroprotective in PD. Maintaining redox balance may be one of the mechanisms underlying silibinin’s neuroprotection. Further study is required to fully understand the potential role of neonatal iron intake and silibinin in aging process and PD and to develop effective therapeutic strategies to slow aging and PD neurodegeneration.

## Conflict of Interest Statement

The authors declare that the research was conducted in the absence of any commercial or financial relationships that could be construed as a potential conflict of interest.
